# Stressful Life Events as a Predictor for Nonsuicidal Self-Injury in Southern Chinese Adolescence: A Cross-Sectional Study

**DOI:** 10.1097/MD.0000000000002637

**Published:** 2016-03-07

**Authors:** Jie Tang, Wei Yang, Niman Isse Ahmed, Ying Ma, Hui-Yan Liu, Jia-Ji Wang, Pei-Xi Wang, Yu-Kai Du, Yi-Zhen Yu

**Affiliations:** From the School of Public Health, Guangzhou Medical University, Guangzhou, Guangdong (JT, PXW, JJW); Department of Nutrition and Food Hygiene, and MOE Key Lab of Environment and Health (WY); Department of Child, Adolescence & Women Health Care, School of Public Health, Tongji Medical College, Huazhong University of Science & Technology, Wuhan, Hubei (NIA, YKD & YZY); and Guangzhou Women and Children Medical Center (YM & HYL), Guangzhou, Guangdong, China.

## Abstract

Stressful life events have been implicated in the etiology of kinds of psychopathology related to nonsuicidal self-injury (NSSI); however, few studies have examined the association between NSSI and stressful life events directly in Chinese school adolescents. In this study, we aim to estimate the prevalence rate of NSSI and examine its association with stressful life events in Southern Chinese adolescents. A total sample of 4405 students with age ranged from 10 to 22 years was randomly selected from 12 schools in 3 cities of Guangdong Province, China. NSSI, stressful life events, self-esteem, emotional management, and coping methods were measured by structured questionnaires. Multinomial logistic regression was used to examine the association of NSSI with stressful life events. Results showed the 1 year self-reported NSSI was 29.2%, with 22.6% engaged in “minor” NSSI (including hitting self, pulling hair, biting self, inserting objects under nails or skin, picking at a wound) and 6.6% in “moderate/sever” NSSI (including cutting/carving, burning, self-tattooing, scraping, and erasing skin). Self-hitting (15.9%), pulling hair out (10.9%), and self-inserting objects under nails or skin picking areas to dram blood (18.3%) were the most frequent types of NSSI among adolescents. Results also showed that “Minor NSSI” was associated with stressful life events on interpersonal, loss and health adaption, and “moderate/severe NSSI” was associated with life events on interpersonal, health adaption in Southern Chinese adolescents, even after adjusted for sex, age, residence, self-esteem, coping style, and emotional management. Results further suggested stressful life events were significantly associated with less risk of NSSI in those who had good emotional management ability.

## INTRODUCTION

Nonsuicidal self-injury (NSSI) refers to the deliberate destruction of one's own body tissues without suicidal intent and for purposes not socially sanctioned.^1^ The common types of NSSI include self-hitting, self-cutting, self-burning, self-banging, and self-scratching, although people who engaged in NSSI usually used multiple types.^2^ NSSI is one of the most familiar behaviors among adolescents with lifetime prevalence rates ranging from 13% to 23.2% and the median age of onset is around 13 years or 14 years among individuals with a history of NSSI.^3–5^

NSSI represents a paramount health issue for adolescents and young adults. It not only directly destructed one's own body tissue, but also it may be associated with numerous psychiatric difficulties, and a range of internalizing and externalizing disorders.^6,7^ There were many studies indicated that NSSI is one of the strongest predictors of suicidal ideation, suicidal attempts, and suicidal completion.^8–11^ Given the current lack of empirically supported prevention and treatment options for NSSI in adolescents, understanding the potential risk factors for NSSI may help identify promising targets for early interventions and future prevention.^12^

Previous studies have documented that stressful life events increase the risk of most types of psychopathology, such as major depressive disorder, impulsive aggression, suicidal ideation and suicidal attempts, and so on.^13,14^ One recent study indicated that people with major depressive disorder who had been exposed to certain stressful life events are at elevated risk for future suicide attempts after accounting for demographic factors and psychiatric comorbidity.^15^ This stress exposure model of psychopathology has been applied to nonsuicidal self-injury recently. For example, some studies indicated that higher rates of stressful life events are associated with greater NSSI.^16–18^

The other theoretical model proposed by Nock also suggested that stressful life events have the similarly prominent role as proximal risk factors for NSSI, as certain individuals would experience certain physiological response when confronted with stressful life events, and may be at risk for engaged in NSSI as a coping strategy particularly among those with difficulties in emotional regulation.^19^ Although these theoretical models support a relation between stressful life events and NSSI, few studies to date have directly examined the degree to which stressful life events lead to NSSI among adolescents, except for research focusing on childhood abuse and other severe early life adversities.^18^

In fact, the association between stressful life events and NSSI is very complex. On one hand, NSSI represents a complex concomitant interplay of psychological, social, and biological factors, and stressful life events are known as a prominent risk factor for NSSI which should be influenced by other risk factors and may be interacted with other factors, such as self-esteem, emotional, management.^20^ Previous study reported that the family factors, such as physical abuse, sexual abuse, and neglect, are the most damaging for child development, and those family risk factors result in long-lasting reduced self-esteem and damaged ability of social, emotional, and cognitive function.^21^ What is more, personal coping style play an important role in the process of dealing with stressful life events, in which positive coping style is generally associated with better stressor resolutions, whereas negative coping is associated with worse results.^22^

On the other hand, the causality of stressful life events and NSSI has inconsistently reported. In some studies, stressful life events interacted with demographical variables to predict NSSI both among inpatient sample and community sample.^17^ While in one study, the frequency of lifetime and past year NSSI predicted the occurrence of interpersonal stressful life events among late adolescents.^23^ Other study even proposed that NSSI may perpetuate a “vicious cycle” of stressful life events,^24^ in which engaged in NSSI could predispose individuals to more negative events in the future life, the occurrence of stressful life events could in turn predispose these individuals to become more vulnerable to the effects of stressors, and consequently, the risk for NSSI becomes greater. Therefore, more researches are needed to better our understanding of the relationship between stressful life events and NSSI.

In this study, we want to use data from a large sample of adolescents from 12 schools in south of China to investigate the epidemiology of NSSI and to examine the relationship between stressful life events and NSSI. Based on the literature review, we hypothesis that stressful life events would be associated with NSSI, even after taking self-esteem, the ability of emotional regulation, and demographic characteristics.

## METHODS

### Study Sites and Participants

This study was conducted from June 2013 to December 2014 in Guangdong Province located in south China. A multistage cluster sampling was carried out to generate a diverse sample. First, Shenzhen, Yangjiang, and Qingyuan cities that represented good, general, and poor socioeconomic status were selected from 21 cities in Guangdong Province. Second, in each selected city, with help of the local education agency, 1 public senior high school and 1 public junior high school that represented the average education level were selected from the rural area and the urban area, respectively. Third, in each of the selected school, 1 class or 2 were randomly selected from each grade. We had classes as the test unit except students who have severe mental disorders (depressive disorder, anxiety disorder, obsession, schizophrenia, etc) and severe parenchymal disease (heart disease, diabetes, hepatitis, pulmonary tuberculosis, etc). Finally, we recruited a sample of 4619 students from 71 classes in those 12 selected schools, and a written consent letter was sent to the students or their guardians. 85 students refused to participate in the study and 23 were absent from the school; hence, the survey included a total sample of 4501students. According to initial screening based on the completeness of the questionnaires, 96 participants were excluded due to incomplete questionnaires. Therefore, the final study population in this study consisted of 4405 students, 2217 boys and 2188 girls, with age ranging from 10 to 22 years. The actual response rate was 95.4% (4405/4619) and all participants were Han nationality.

The self-administered survey was anonymous, and it was completed in classroom during 30 to 45 min. There were 1 or 2 trained staffs who explained the purpose and procedures of the study on each classroom. The participants were told that the survey did not represent a test and that they should place emphasis on answering the question honestly and accurately. The participants were also promised that all the information included in the survey will be kept confidential and will only be used for scientific research. The same announcements were also shown in the front of questionnaire.

### Ethics Statement

The Ethical Committee of the Medical Association of Guangzhou Medical University approved the proposal of the study. The questionnaires were approved by the target school and a written informed consent was obtained from the parents or their guardians of each participant.

### Instruments

#### Nonsuicidal Self-Injury

NSSI of the participants over the previous 12 months before the survey were assessed by the Function of Self-Mutilation (FASM),^25^ which was widely used in clinical interview and community sample of adolescents with acceptable psychometric properties.^26,27^ It presented in checklist format that evaluate the behavioral functions and the frequency of different types of NSSI; however, in the present study, we only measured the frequency of different methods of NSSI. The FASM include 10 different behaviors yielded 2 factors: “moderate/severe NSSI” and “minor NSSI,”^25^ the former included cutting/carving, burning, self-tattooing, scraping, and erasing skin, which considered more clinically severe in nature, whereas the latter included self-hitting, pulling hair, self-inserting objects under nails or skin picking areas to dram blood and picking at a wound, which was less severe. The internal consistency for moderate/severe and minor NSSI in the present study was 0.76 and 0.81.

#### Adolescent Self-Rating Life Events Check List

Adolescent Self-Rating Life Events Check List (ASLEC) was developed by Liu XC in 1987, which included 27 items.^28^ The scale measured 6 factors related to life events: interpersonal relationship factor (IRF), academic factor (AF), punishment factor (PF), loss factor (LF), health adaption factor (HAF), and other factor (OF). Participants rated “how the item negatively affected you during the last 12 months” by using 5-point Likert scale ranging from 1 (not at all) to 5 (a great deal), or indicated the event had not happened to him/her (not applicable). Higher total scores on the scale represent a greater impaction of life events experienced in the last 12 months. According to Liu XC's interpretation of ASLEC, the score of subscale were group into 5 groups: have no effect, mild, moderate, severe and extremely severe, as described in detailed elsewhere.^28^ To better understand the association between stressful life events and NSSI, we categorized the 5 groups into 2 levels: no effect (include “have no effect” and “mild”) and have effect (include “moderate,” “severe,” and “extremely severe”). The ASLEC has been reported to have good psychometric properties (internal consistency was 0.849).^29^ The Cronbach's α within the present sample was 0.91.

#### Emotional Management Ability

The emotional management ability was assessed by a subscale of the Emotional Intelligence Inventory (EII).^30^ The subscale includes 4 items, and each one rated on 4-point Likert scale ranging from 1 (always like this) to 4 (never like this), the total of scores were categorized into 3 levels: good (X > M + SD), average (M+SD ≥ X ≥ M-1SD), and poor (X < M − 1SD).^31^ It had acceptable internal consistency (α = 0.79) in the present study.

#### Trait Coping Style

The Chinese Trait Coping method questionnaire was used to assess positive and negative coping strategies of the participants, as the coping method is a very important mediator of emotional regulation.^32^ The questionnaire has 2 subscales: negative coping and positive coping, each subscale has 10 items, rated from 1 to 5, the higher scores of the subscale the greater intensity of related coping style. In the present study, the Cronbach's α values for positive and negative coping style were 0.854 and 0.843, respectively.

#### Self-Esteem

The Chinese version of Rosenberg's Self-Esteem Scale^33^ was used to measure the level of self-esteem among the participants. The instrument consisted of 10 items rated on a 4-point Likert scale from “1 = strongly disagree” to “4 = strongly agree.” Items numbered 3, 5, 8, 9, and 10 are reverse scored, the higher the scores the higher the self-esteem. Cronbach's α of this instrument was 0.79 in the previous study,^34^ and its value in this study was 0.74.

Other confounding variables: we used an additional questionnaire to collect the potential confounding variables—residence (urban/rural), 1 child family (yes/no), parents’ highest education (college/senior high school/junior high school), family structure (extend or nuclear family/step family/single-parent family/grandparent family/others), and so on. This additional questionnaire has been proofed to have good reliability and validity.

### Statistical Analysis

Statistical analyses were conducted by SPSS for windows 17.0 (SPSS Inc., Chicago, IL). Mean, standard deviation, or percentage was used to describe the characteristics of the study participants. *T-*tests were used to compare continuous data and chi-square tests were used to compare frequencies data. Multinomial logistic regression was used to examine the association between NSSI and stressful life events. Both odds ratios and 95% confidence intervals (95% CI) for potentially confounding effects of the stressful life events, emotional management, trait coping method, self-esteem, and demographic variables (included gender, age, family and school environment, and so on) were reported. The significance level was set at 0.05, and all tests were 2-sided. To better understand the association between stressful life events and NSSI, we transferred the score of ASLEC and EM into categorical variable in the multinomial logistic regression.

## RESULTS

### Overview

A total sample of 4405 students was finally analyzed in the present study. Table 1 showed the demographic characteristics and the related variables of the participants. The self-reported 1-year prevalence rate of NSSI was 29.2%, 22.6% participants reported “minor NSSI” and 6.6% reported “moderate/sever NSSI,” respectively. Gender difference on the prevalence rate of NSSI was significant (χ^2^ = 10.473, *P* = 0.005). Gender difference on the loss factor (LF) was insignificant (*t* = 0.984, *P* = 0.325), whereas gender differences on interpersonal relationship factor (IRF), academic factor (AF), punishment factor (PF), health adaption factor (HAF), other factor (OF), and total scores of ASLEC were significant ([*t*_1_ = 2.408, *P*_1_ = 0.016]; [*t*_2_ = −4.017, *P*_2_ < 0.001]; [*t*_3_ = 6.150, *P*_3_ < 0.001]; [*t*_4_ = −2.467, *P*_4_ = 0.013]; [*t*_5_ = 10.777, *P*_5_ < 0.001]; [*t*_6_ = 3.169, *P*_6_ = 0.002]). Boys had greater ability of emotional management than girls (*t* = 4.920, *P* < 0.001), whereas girls had higher self-esteem than boys (*t* = 4.292, *P* < 0.001).

**TABLE 1:**
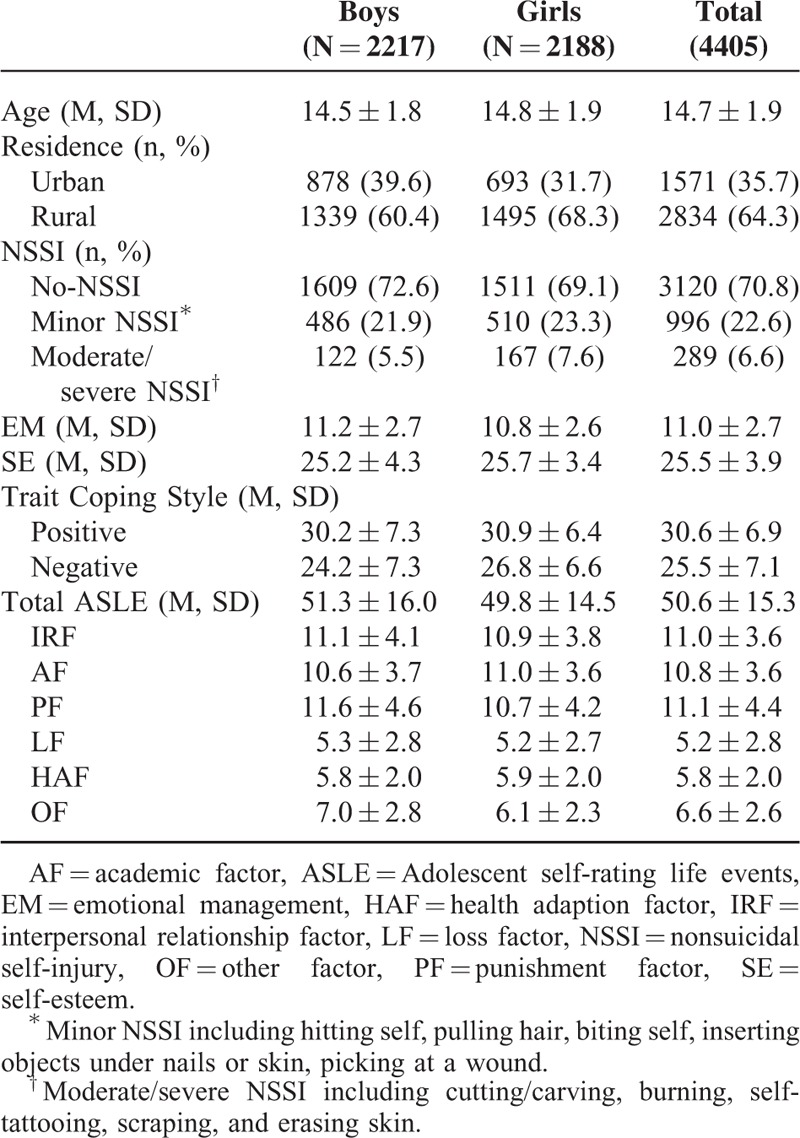
Demographic Characteristics and Related Variables of Study Participants

### Descriptive Characteristic of NSSI Among Study Population

Among the 1285 adolescents who had engaged in NSSI during the past year, 557 (43.3%) reported NSSI 1 to 4 times, 273 (21.2%) reported 5 to 10 times and 455 (35.5%) reported NSSI ≥11 times. Regarding types of NSSI reported in the sample, 98.4% of the self-injuries reported engaged in 1 to 5 types of NSSI, the mean number of types of NSSI performed was 1.9 (SD = 1.2, median = 2, mode = 1.0, range = 1–8). Self-hitting (15.9%), pulling hair out (10.9%), and self-inserting objects under nails or skin picking areas to dram blood (18.3%), were the most frequent NSSI behaviors in the study population.

### Association Between NSSI and Stressful Life Events

Gender difference on occurrence rates of NSSI was existed; thus we examined the association of NSSI and ASLEC stratified by the gender. The results are showed in Table 2. Self-reported NSSI were significantly associated with ASLE and each dimension of ASLE in both boys and girls. The participants who reported NSSI during the past 12 months before investigation were more likely to experience stressful life events. NSSI were also significantly associated with emotional management, self-esteem, and coping method as showed in Table 2.

**TABLE 2:**
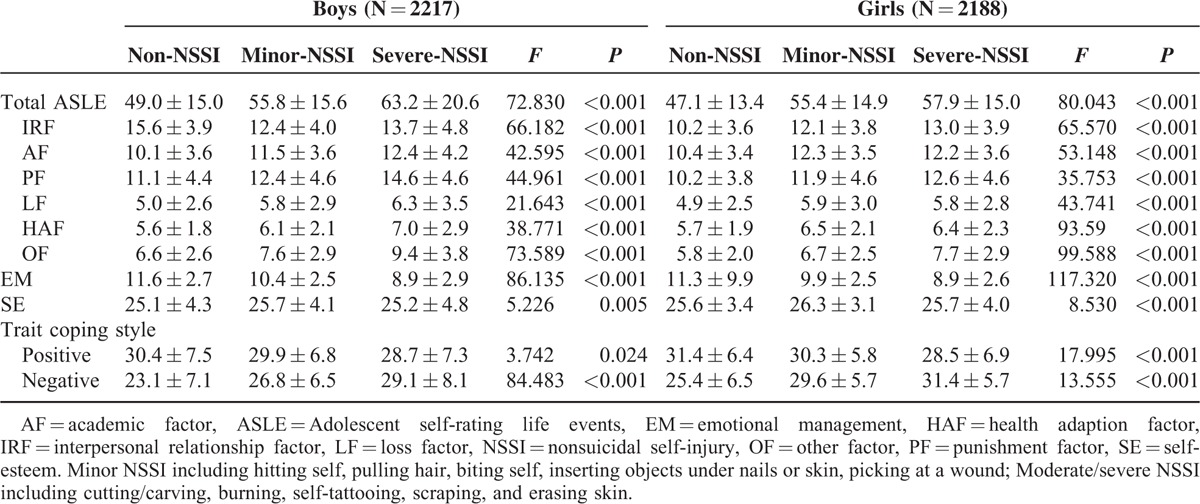
Scores for ASLEC With Reported NSSI Stratified by Gender

### Association Between NSSI and Stressful Life Events After Controlling Confound Variables

Multinomial logistic regression analysis was conducted to examine the association between NSSI and stressful life events controlling for age, gender, residence, family economic status, and other potential confounding variables. Results are showed in Table 3. Model I showed that IRF, AF, LF, HAF were significantly associated with “minor NSSI” after controlling for gender, age, and other demographic characteristics (OR_IRF_ = 1.73 [1.42–2.10], OR_AF_ = 1.41 [1.15–1.73], OR_LF_ = 1.25 [1.02–1.52], OR_HAF_ = 1.38 [1.06–1.45]); IRF, HAF, and OF were significantly associated with “moderate/severe NSSI” after controlling for potential confounding variables (OR_IRF_ = 2.19 [1.62–2.95], OR_HAF_ = 1.38 [1.01–1.89], OR_OF_ = 3.07 [1.23–4.91]). No significance between LF and NSSI was found.

**TABLE 3:**
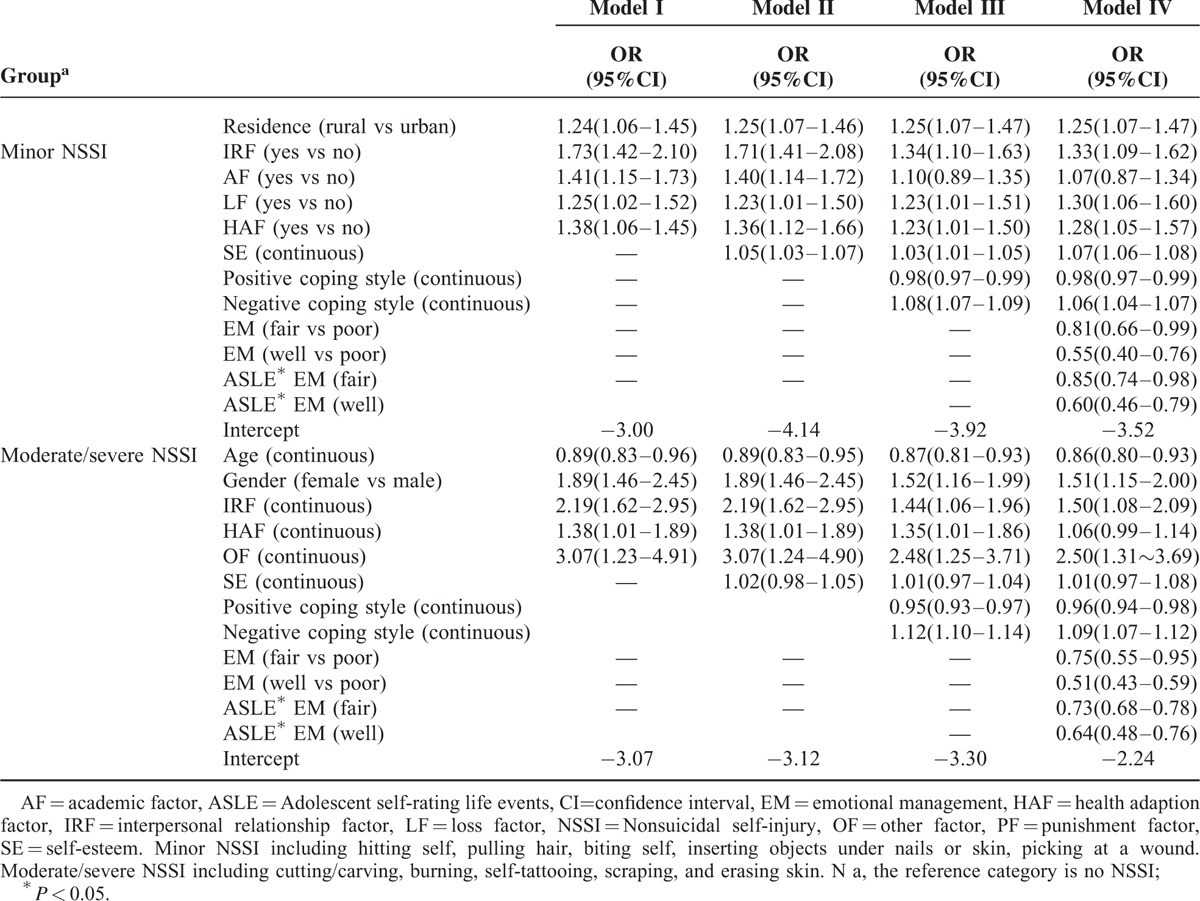
Multinomial Logistic Regression Model for Predicting NSSI

We conducted a multinomial logistic analysis to examined whether self-esteem or/and coping style could change the association between NSSI and stressful life events, the results (Table 3, Model II, Model III) showed that the association between IRF, AF, LF, HAF and “minor NSSI” remained (OR_IRF_ = 1.71[1.41–2.08]/1.34[1.10–1.63], OR_AF_ = 1.40[1.14–1.72]/1.10[0.89–1.35], OR_LF_ = 1.23[1.01–1.50]/1.23[1.01–1.50], OR_HAF_ = 1.36[1.12–1.66]/1.23[1.01–1.50]). The significant association between IRF, HAF, OF, and “moderate/severe NSSI” also existed (OR_IRF_ = 2.19[1.62–2.95]/1.44[1.06–1.96], OR_HAF_ = 1.38[1.01–1.89]/1.35[1.01–1.86], OR_OF_ = 3.07[1.24–4.90]/2.48[1.25–3.71]).

Emotional management and its interaction with stressful life events may have effect on the association between NSSI and stressful life events. Therefore, we included emotional management and its interaction with stressful life events into Model IV. The results illustrated that the association between NSSI and stressful life events still existed (for “minor NSSI”: OR_IRF_ = 133 [1.09–1.62], OR_AF_ = 1.07[0.87–1.34], OR_LF_ = 1.30[1.06–1.60], OR_HAF_ = 1.28[1.05–1.57]; for “moderate/severe NSSI”: OR_IRF_ = 1.50[1.08–2.09], OR_HAF_ = 1.06[0.99–1.14], OR_OF_ = 2.50[1.31–3.69]). The interaction of emotional management and stressful life events was significant and negative (for “minor NSSI”: β = −0.16, SE = 0.07; for “moderate/severe NSSI”: β = −0.31, SE = 0.09), suggesting that stressful life events was associated with a greater risk of NSSI among those who had poor level of emotional management than who had average or good level of emotional management.

## DISCUSSION

In the present study, we investigated the epidemiology of NSSI and explored its association with stressful life events based on a sample of Chinese school students. The results revealed that ∼29.2% of the sample engaged in NSSI during the past year before the survey, and there was evidence indicated that “minor NSSI” was associated with stressful life events on interpersonal, loss and health adaption, and “moderate/severe NSSI” was associated with life events on interpersonal, health adaption even after adjusted for sex, age, residence, self-esteem, coping method, and emotional management.

NSSI was not to be considered as an illness in community adolescent until the American Psychiatric Association incorporated NSSI into the DSM-V in 2013,^35^ which may partly explain the rising prevalence of NSSI around the world.^36^ An overview of 50 studies from 2005 to 2010 revealed an average lifetime prevalence rate of 18%.^4^ It is estimated that the prevalence of at least 1 past wounding among adolescents even up to 34%.^37^ In the present study, the prevalence rate of 1 year NSSI was 29.2%, with 22.6% engaged in “minor NSSI” and 6.6% engaged in “moderate/severe NSSI,” which was similar with the result reported by You among Chinese students.^13^ Previous studies indicated the prevalence rates of NSSI between men and women during preadolescence and adolescents were similar;^38,39^ however, the prevalence rates of NSSI in this study showed a significant gender difference. The epidemiology of NSSI varied with each studies not only due to the heterogeneity of the study participants, but also correlate to its measurement instruments. Therefore, in order to obtain comparable epidemiological data of NSSI across different studies, standard survey methods and measurement instruments are necessary to develop. The present study will provide important information for health professionals, such as pediatricians, primary care physicians, and school physicians, and other societies. And also create awareness that NSSI is becoming a paramount health issue for adolescents and thus they need to be equipped with up-to-date NSSI knowledge and intervention technology.^40,41^

The present study showed that stressful life events were associated with risk for NSSI in adolescents, which were similar with previous studies focusing on early life adversities or stressful life events in relation to self-injury behaviors.^16,17^ Additionally, the results also showed that stressful life events combined to emotional management to exert an interactive role on NSSI, with less NSSI reported in those who have well of emotional management, which also consistent with previous study.^42^ More importantly, the present study found that the effects of stressful life events on NSSI were more subtle: “minor NSSI” was associated with stressful life events on interpersonal, loss and health adaption, and “moderate/severe NSSI” was associated with life events on interpersonal, health adaption even after adjusted for sex, age, residence, self-esteem, coping strategy, and emotional management. Thus, this study adds to the literature demonstrating that different types of stressful life events may different effects on NSSI among Chinese adolescents.

The observed association between stressful life events and NSSI could be interpreted from the following perspectives. First, according to the construction of ASLEC, interpersonal include “misunderstood, discriminated, family neglect, and/or abuse.”^30^ Stressful life events on interpersonal not only associated with distrust of others, poor coping method, but also associated with poor emotional management and lack of social supports, and all of those have been known as risk factors for NSSI.^43–45^

Second, according to the stress-diathesis model, stressful life events could act as a diathesis that could lead to depression symptom and hopelessness, which had been associated with NSSI and suicidal behaviors.^46–48^ However, not all kind of stressors will lead to unwanted affective experiences among adolescents. The previous study indicated that life events on loss and interpersonal were the most serious stressors among youth.^49^ In addition, the effect of stressful life events were not only depends on the type of stressors, but also relates to their frequencies, namely, the effects of stressful life events can be accumulated during a given period. It had reported that stressors on academic and interpersonal were the most common stressors among adolescents.^50^ You and his colleagues also reported that among all stressful life events only interpersonal stressors, poor school performance were link with suicidal behaviors.^51^

Moreover, emotional regulation is an important mediator in association between stressful life events and NSSI, which refers to the process of individual management and stress cognition.^52^ Adolescence is a period when physical and psychological development are not mature; thus, the ability of self-control and stress cognition are limited, adolescents cannot cope all the stress from life events, once stress outstrips one's emotional regulation ability, and NSSI may be commonly carried out to compensate for emotional regulation.^53^ It is worth mentioning that emotional regulation ability become much greater with growing age,^54^ and adolescents with greater emotional management ability always develop skills for controlling one's negative emotion.^55^

The present study was interpreted in light of several limitations. First, although the study achieved a relatively large sample size, further research to replicate findings among a more representative sample is warranted. In our sample, the age of the participants ranged from 10 to 22 years, which may incorporate into preadolescents and youth adults, thus reduced the authenticity of the research.^56^ Second, data collection in our study were based on retrospective self-reported, which limited our findings due to potential reporting inaccuracies and misinterpretation of questions by participants. Third, the present study did not include all the factors when assessing the association between stressful life events and NSSI, those included subjects’ psychological factors (anxiety, depression, etc), impulsivity and social support and so on, which may led to an underestimate of strength of the association. In addition, the cross-sectional nature of the serial mediation analyses precludes conclusions regarding the precise temporal relation of the proposed mediators and outcomes. All those limitations of the present study remain key direction for our further study.

In conclusion, the prevalence rate of NSSI is high among Chinese students; stressful life events may be very important risk factors of NSSI. However, the result should be validated in further study, especially in the prospective cohort study.
